# Research impact assessment of a Canadian digital health funding program: a case study

**DOI:** 10.1186/s12961-025-01356-2

**Published:** 2025-06-23

**Authors:** Jessica Nadigel, Bahar Kasaai, Halla Thorsteinsdóttir, Susan Rogers, Meghan McMahon, R. Jane Rylett, Richard H. Glazier

**Affiliations:** 1 CIHR Institute of Health Services and Policy Research, ICES, 2075 Bayview Avenue, Toronto, ON M4N 3M5 Canada; 2 CIHR Institute of Population and Public Health, Université de Montréal, 7077 Avenue du Parc, Suite 2043, Montreal, QC H3N 1X7 Canada; 3 Institute of Health Policy, Management and Evaluation, University of Toronto, 155 College Street, Suite 425, Toronto, ON M5T 3M6 Canada; 4 Small Globe Inc, Toronto, ON Canada; 5 CIHR Institute of Aging, Western University, 1151 Richmond Street North, London, ON N6A 5K8 Canada; 6 Department of Physiology and Pharmacology, Schulich School of Medicine & Dentistry, Western University, 1151 Richmond Street North, London, ON N6A 5K8 Canada; 7 Institute for Clinical Evaluative Sciences (ICES), V Wing, 2075 Bayview Avenue, Toronto, ON V1-06, M4N 3M5 Canada

**Keywords:** Digital health, eHealth, Research impact, Evaluation, Research funding

## Abstract

**Background:**

Digital innovations have the potential to enhance equitable access to health systems, improve care integration and support learning health systems. Research funders make substantial investments in digital health research to advance the uptake of evidence-informed digital solutions within health systems, yet their impacts on health and health system outcomes, health equity, policy and practice remain poorly understood. Research impact assessments (RIAs) serve as a vital tool for funders to examine the links between research investments and real-world change. The Canadian Institutes of Health Research commissioned an RIA on its largest digital health program, the eHealth Innovations Partnership Program (eHIPP), to understand the program’s outputs and impacts.

**Methods:**

This study applied two complementary frameworks, the Canadian Academy of Heath Science’s (CAHS) Making an Impact Framework and the Canadian Health Services and Policy Research Alliance’s (CHSPRA) Informing Decision-Making Framework, to assess the research impact of the eHIPP program, funded from 2015 to 2021. A mixed-methods approach was taken to collect and analyse data from eHIPP grant recipients and their partners.

**Results:**

The eHIPP program supported 22 research teams through a total investment of CAD$ 42M. The RIA revealed impacts in the areas of capacity development, knowledge creation, informing decision-making and health outcomes. The teams generated 36 co-designed, evidenced-informed solutions, 79 publications, 194 presentations and 38 media interviews or articles. Solutions were reported to influence health system practice (52%) and policy (33%), improve health outcomes (62%), enhance equitable access to care (62%), improve patient (62%) and provider experience (52%), increase cost-effectiveness (52%), enhance population health (48%) and improve health equity (43%).

**Conclusions:**

This RIA study highlights the importance of stakeholder collaboration, robust partnerships and co-design approaches in effectively integrating patient-centred digital health solutions into health systems. These elements are key to advancing the Quintuple Aim (improved cost, population health and equity and experience of patients and providers) and supporting evidence-informed decisions. This paper presents a first case study applying the CAHS and CHSPRA frameworks to assess the impacts of a large digital health funding program. Further, it explores the program’s outcomes and impacts and highlights considerations, successes and challenges for funders when applying RIA.

**Supplementary Information:**

The online version contains supplementary material available at 10.1186/s12961-025-01356-2.

## Background

Canada is facing challenges in delivering equitable and coordinated care to patients, grappling with poor value for money and subpar health system performance relative to other comparable health systems in high-income countries [1]. Concurrently, the ongoing advancement of research and progress in digital health and data science offers a timely window of opportunity to harness digital technology as one lever to enhance health system performance. Evidence-informed digital innovations have the potential to improve equitable access to health systems for patients, enhance the capacity of the health system to integrate care across the care continuum, enable interconnectivity across all levels (patients, providers, health system databases) and foster learning health systems for continuous learning and improvement, resulting in greater cost-effectiveness of service delivery and overall care for patients and population groups [2, 3]. However, with market-driven expansion in the digital health space, certain risks need to be mitigated. Governments and institutions must establish robust data privacy and governance policies and appropriate evidence-based digital health standards [3, 4] to protect patient privacy and prevent the uptake of unproven, unsafe or inappropriate solutions into the health system. Furthermore, health systems must deal with inequitable access to digital solutions and health services for communities who are already underserved [5].

Research funding agencies invest in digital health research to address risks and promote the development and adoption of equitable, evidence-informed digital solutions by health systems. From 2013 to 2023, the Canadian Institutes of Health Research (CIHR), Canada’s federal health research funding agency, supported 420 digital health-related projects, for a total investment of CAD$ 95.6 million [6]. However, little is known about the impacts of these investments and whether evidence generated from these research projects influenced policy, practice or health outcomes. Research impact assessment (RIA) is essential for evaluating the effectiveness of research and the value of research investments. It enables funders to understand the nature and dynamics of whether and how research and innovations are integrated into health systems and influence policy and practice decisions that will potentially lead to improved population health. It also enables funders to improve the design of future programs. The field of RIA is gaining traction and there is growing recognition that research funders, institutions and organizations should expand their focus beyond traditional academic metrics [7–9] (e.g. number of grants, publications, citations, journal impact factors) to broader health, societal and economic impacts, including influence on policy and practice, engagement and partnership across sectors and stakeholders and training and mentorship. In its quest to better understand, capture and illustrate the impact of its digital health research investments, CIHR undertook an RIA on its largest digital health funding program – the eHealth Innovations Partnership Program (eHIPP).

### The eHealth Innovations Partnership Program (eHIPP)

The eHealth Innovations Initiative was launched in 2012 by CIHR’s Institute of Health Services and Policy Research (IHSPR) and Institute of Aging (IA), in collaboration with CIHR’s Institute of Neuroscience, Mental Health and Addictions (INMHA) and Institute of Human Development, Child and Youth Health (IHDCYH). The initiative aimed to stimulate the design, testing, evaluation and spread of evidence-based eHealth solutions that are grounded in the needs of patient groups and populations [10]. The initiative emphasized partnerships across various stakeholders, with the ultimate goals of improving the quality of outcomes and the cost-effectiveness of patient-centred care and strengthen Canada’s competitive standing in the digital health sector. The eHealth Innovations Initiative’s flagship program, eHealth Innovations Partnership Program (eHIPP), was launched in 2014 with two main goals. The first was to identify patient-oriented eHealth solutions that will improve health outcomes, patient experience and lower the cost of care. The second was to foster partnerships between innovation communities (healthcare providers, patients, families, researchers, health system decision- and policymakers and health technology partners) to co-design eHealth-enabled service delivery programs and evaluate the clinical benefit and cost-effectiveness of the eHealth solutions. The innovation communities were a key program design element, ensuring that the perspectives and needs of all stakeholders were integrated into the development and evaluation processes, ultimately aiming enhancing the relevance, accessibility, sustainability and impact of the eHealth solutions. The eHIPP prioritized two key areas of focus for the innovation communities: (1) early identification of, and intervention for, youth and adolescents (11–25 years of age) with mental health conditions and (2) support of seniors with complex care needs in their homes.

### eHIPP impact assessment

A decade after the launch of eHIPP, the program leads (CIHR’s IHSPR and IA) sought to understand the long-term impacts of the program and whether the program attained its core objectives. This study explores the key outcomes and impacts of the program, the various conditions that may have contributed to its success (external and internal factors, program design elements) and how funding agencies can further support RIA.

**Fig. 1:**
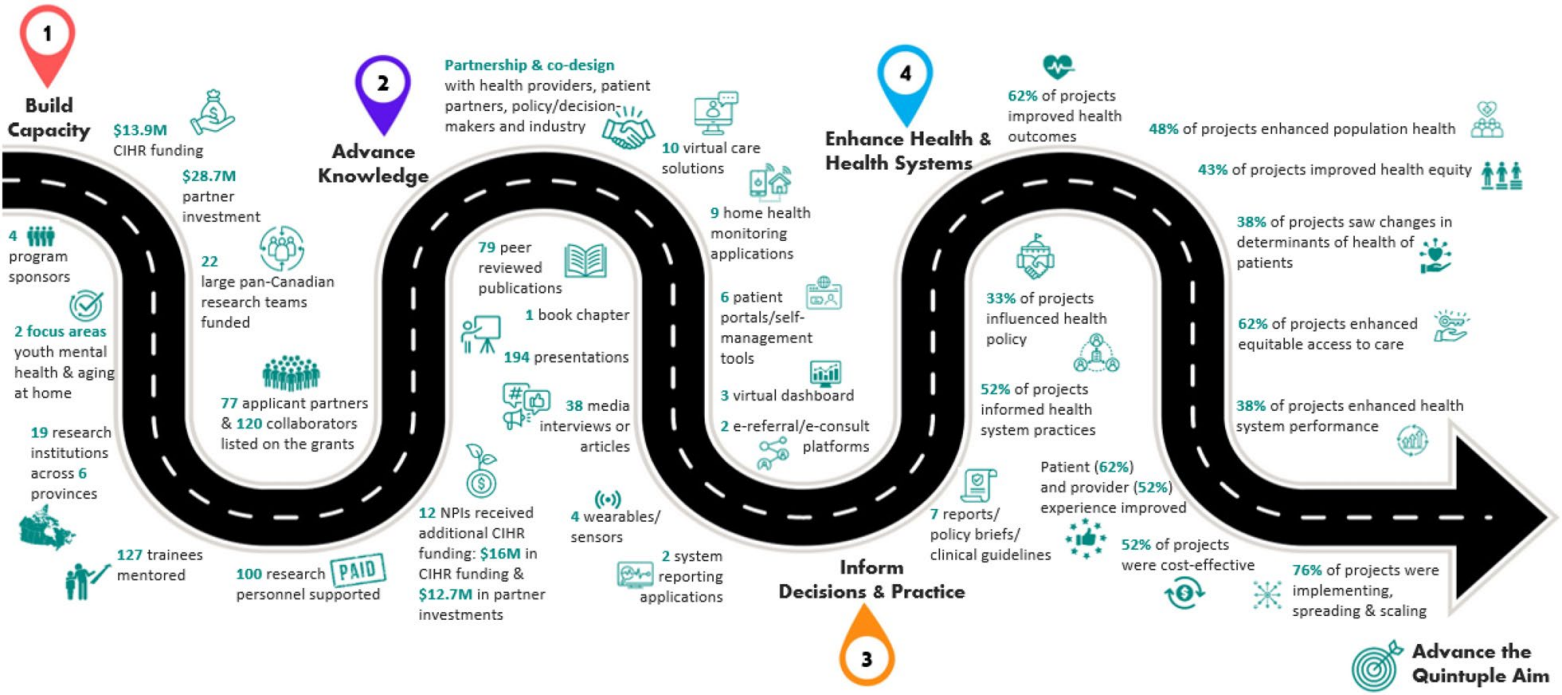
Schema of eHIPP Pathway to Impact, aligned to CAHS & CHSPRA impact categories

To achieve these aims, two complementary impact frameworks that describe the categories, pathways and indicators of research impact were used: the Canadian Academy of Heath Science (CAHS) Making an Impact Framework [11] and the Canadian Health Services and Policy Research Alliance (CHSPRA) Making an Impact: Informing Decision-Making Framework [12]. The CAHS framework, released in 2009, is grounded in the Buxton and Hanney “payback model” [13] and is designed to assess the return on investment in health research. The framework was built to support impact assessment of the various types of health research (biomedical, clinical, health services and systems and population health) and allows for the selection of appropriate metrics and indicators on the basis of the type of health research being assessed. The CHSPRA framework builds upon the CAHS framework to understand the outcomes and impacts of health services and policy research (HSPR) investments, with a particular focus on how HSPR informs decision-making (one of the five key impact domains in the CAHS framework). CIHR-IHSPR has integrated and adapted the CAHS and CHSPRA frameworks to develop a conceptual model to assess the research impact of HSPR funding programs, and has successfully applied this conceptual model to multiple IHSPR programs [14–16]. The model incorporates four key impact categories: (1) capacity building; (2) advancing knowledge; (3) informing decision-making and practice; and (4) enhancing health and health system outcomes. Each impact category contains subcategories and indicators to provide a more detailed characterization of impacts. Figure 1 illustrates the pathway to impact for this RIA study of eHIPP, emphasizing the nonlinear nature of the relationship between research investment and impact. This is the first study to use the CAHS and CHSPRA frameworks as a basis to understand impacts resulting from a large pan-Canadian investment in digital health research.

## Methods

### Study sample and design

RIA is a nascent field that uses a wide range of mixed-methods, often on the basis of the systems, datasets and infrastructure feasible and available to funders [17, 18].

The data for this study were collected between February and April 2023 from eHIPP grant recipients and their partners using a mixed-methods design and sequential-explanatory approach to first collect and analyse quantitative data (surveys) and then qualitative data (follow-up interviews).

The study sample eligibility criteria include eHIPP research teams (also known as the innovation community) who received a grant during the funding period of 2015–2021. Each research team was composed of a lead investigator, and at least one representative of each of the following stakeholder types: industry partner, a clinical lead, decision/policymaker and patient/family representative.

### Document analysis

The methods involved a comprehensive review of CIHR documents related to the program, including descriptive information about the eHIPP program [10], the eHIPP funding opportunity text [19], information from CIHR’s funded project databases and the final reports submitted by the lead investigators (Additional File 1).

### Focus group, surveys and informant interviews

The program architects were invited to a focus group discussion to unpack the motivation, design elements and expected outputs for the program (*n* = 2 former scientific directors, *n* = 2 current scientific directors; *n* = 2 associate scientific directors).

An online survey was developed to probe the lead investigators’ views on the impacts of their research and the value of their partnerships. The online survey questions were informed and shaped by the CAHS and CHSPRA impact frameworks, the objectives of both the eHIPP program and the impact assessment, the review of documents and the initial focus group consultation. The Quintuple Aim (improved population health outcomes, better patient and provider experience, improved value for money and enhanced health equity) [20, 21] is widely regarded as the guiding principles for advancing health system improvement and is increasingly used in Canada as a framework to assess health system performance [22, 23]. To align with current best practices, the study team grounded the health- and health-system-related impact questions in the study survey within an expanded interpretation of the Quintuple Aim principles, which also included changes in determinants of health, equitable access to care and health system performance. Survey responses were considered to have positive impacts when respondents selected the top two ratings on the Likert scale.

The survey (Additional file 2) was sent to the lead investigators of the 22 funded eHIPP projects (Additional file 3) in their language of choice (English or French) in February 2023, 2 years after the end of grant term. The lead investigators were asked to share an anonymized version of the survey with their project partners to garner their feedback. In total, 21 completed survey responses were received, 12 of which were attributed to lead investigators and 9 anonymous responses.

To further explore the perspectives of the research teams, 13 semistructured interviews (Additional file 4) with lead investigators (*n* = 4) and their project partners, including industry representatives (*n* = 2), patients (*n* = 2), healthcare providers (*n* = 2) and policy/decision-makers (*n* = 3) were conducted. The selection of interviewees was guided by considerations of the digital health focus area, stakeholder type and geographic region to ensure diversity of perspectives. Interviews were conducted virtually via Zoom from March to April 2023. With participants’ consent, discussions were recorded and audio recordings were transcribed verbatim.

### Data analysis

Quantitative survey and final report data were first analysed and used to inform the approach, questions and key themes discussed in the semistructured interviews with eHIPP team members. CIHR commissioned a third party, Small Globe Inc, to support the design, data collection, thematic analysis and interpretation of interviews, focus group discussions and surveys, with iterative and strategic input from study authors.

## Results

### Pathways to impacts: from research investment to evidence-informed digital health innovations and health system improvements

The objectives of eHIPP aligned well with the CAHS and CHSPRA frameworks’ pathways to impact. Namely, the theory of change for the funding program was intentionally designed to: (1) direct targeted research investments; (2) advance the evidence base and build capacity (e.g. facilitated by intersectoral partnerships between health system players, researchers, healthcare providers, industry and patients/families/public); (3) design innovative, evidence-informed eHealth solutions for prioritized populations; and (4) enable their eventual uptake and implementation in healthcare delivery settings. In turn, (5) coupling these eHealth solutions with knowledge mobilization strategies (e.g. through their partnerships and the integration of evidence into policy and practice), can result in (6) advancing the Quintuple Aim principles of improved health and health system outcomes, enhanced patient and provider experience, improved cost-effectiveness of patient-centred care, enhanced health equity and overall better population health [20, 21].

Table 1 represents the broad CAHS/CHSPRA impact categories, subcategories and eHIPP metrics used in this RIA. The following sections expand on each impact category.

**Table 1:** CAHS & CHSPRA impact categories and eHIPP metrics

Category	Subcategory	Metric
Build capacity	Investments	CAD$ 13.9 million CIHR investment
		CAD$ 28.7 million partner investments (CAD$ 2.9 million cash; CAD$ 25.9 million in-kind)
	Program sponsors	4 program sponsors
	Focus areas	2 focus areas: (1) early identification of, and intervention for, youth and adolescents with mental health conditions; (2) support of seniors with complex care needs in their homes
	Research teams	22 large pan-Canadian research teams funded
		19 research institutions across 6 provinces
		77 applicant partners and 120 collaborators
	Training	127 trainees were supervised and mentored
	Career support	100 research personnel supported through the grants
	Future funding	20 lead investigators participated in subsequent CIHR operating grant funding
		12 lead investigators received additional CIHR funding (CAD$ 16 million in CIHR funding and CAD$ 12.7 million in partner investments)
Advance knowledge	Innovations	36 evidence-informed co-designed solutions
	Publications	79 peer reviewed publications
	Presentations	194 presentations
	Media	38 media interviews or articles
Inform decisions and practice	Influence	52% of respondents indicated their solution(s) informed health system practice
		33% of respondents indicated their solution(s) informed health policy
		7 reports/policy briefs/clinical guidelines published
Enhance health and health systems	Improve health	62% of respondents reported their solution(s) improved health outcomes
		48% of respondents reported their solution(s) improved population health
		43% of respondents reported their solution(s) improved health equity
		38% of respondents reported their solution(s) changed determinants of health
	Improve health systems	62% of respondents reported their solution(s) improved equitable access to care
		62% of respondents reported their solution(s) improved patient experience
		52% of respondents reported their solution(s) improved provider experience
		52% of respondents reported their solution(s) improved cost-effectiveness
		38% of respondents reported their solution(s) improved health system performance
	Spread and scale	76% of respondents indicated they were at the stage of either implementing their solution or spreading and/or scaling it
		Of these, 31% reported they were spreading their solution within their organization, 81% reported spreading and scaling their solution beyond their organization within their province/territory and/or to other regions across Canada and 19% reported that their intervention was spread and/or scaled internationally

### Build capacity

#### eHIPP investments and enabling conditions for success

In the focus group discussions, the program architects underscored that the driving force behind eHIPP was to enhance awareness about digital health, given the rising demand for health services and the inability of the health system to respond adequately with in-person care and services. As one focus group participant noted: “there’s no way there will be enough providers to provide the home care, and there will be an obligation to go to digital monitoring support, potentially tele consultation and things like that”. Program architects sought to transform the research landscape by engaging patients and end-users, whilst also fostering partnerships amongst researchers, industry players and other stakeholders. The vision was to foster a more collaborative and integrated eHealth environment in the country, which although time-consuming, would gradually shift the cultural paradigms in the field. The program was structured with four essential design elements to maximize the conditions for success and institute cultural shifts: (1) co-design principles enabled by partnerships and the creation of healthcare innovation communities; (2) person-oriented rather than technology-oriented solutions; (3) engagement, commitment and resources from small- and medium-sized digital health enterprises (to foster a dynamic digital health ecosystem in Canada); and (4) rigorous methods and metrics applied to evaluate costs and help ensure that the consequences and benefits of the research align with patient, provider and healthcare system needs.

To foster commitment from partners, the eHIPP funding design required 1:1 matched funding from applicant-level project partners. The initial CAD$ 13.9 million CIHR investment was leveraged into an additional CAD$ 28.7 million investment (CAD$ 2.9 million in cash and CAD$ 25.9 million in in-kind support) from 77 applicant-level partners. Additionally, there were 120 collaborating organizations from across Canada who endorsed the teams through letters of support. Applicant-level partners and collaborating organizations primarily consisted of for-profit digital health companies, universities/research institutions, provincial health services/system organizations, charities/foundations, ministries of health/health authorities and provincial research funders. Overall, this approach resulted in enhanced national capacity, with a total investment of CAD$ 42.6M and the engagement and commitment of more than 19 research institutions across 6 provinces where research teams were located. 

#### Research team level – eHealth innovation communities

The funding program required the creation of research teams or “ehealth innovation communities”, which advance the “capacity building” impact category of the CAHS and CHSPRA impact frameworks and align to our theory of change (identified above). These research teams were composed of a minimum of one: scientific lead (an independent researcher); clinical lead (a healthcare professional engaged in healthcare delivery); patient end-user (or family/caregiver/member of the public); a health system policy/decision-maker with authority and agency in the local adoption and scale-up of the eHealth innovation; and an industry partner with the resources, commitment, and/or technology to test, implement and scale the promising co-designed digital health innovation.

#### Individual level – Research and trainees

eHIPP had positive impacts on the individual research programs, research teams and careers of lead investigators. A total of 95% of respondents agreed or strongly agreed with the survey statement: “The eHIPP funding has been essential for my research work and my advancements in digital health over the last few years”. Final reporting data indicate that researchers also built and promoted the academic research profiles of others through the mentorship and supervision more than 127 trainees and the support of more than 100 research personnel.

“Overall, I think this grant was really important to me, because it helped launch this amazing thing that we’ve created… And I feel like, none of the experiences that I had good or bad, was a waste. I feel like it led to something really great… So, but without that funding that would never have happened” (Lead investigator).

Since receiving their eHIPP funding, 91% of the lead investigators have participated in subsequent operating grant funding from CIHR, and 12 of these leads have led a total of 28 operating grants, combined. Additionally, 3 lead investigators currently hold Canada Research Chairs [24], 10 are recognized as supervisors on CIHR training awards and 5 have been awarded travel grants or prize funding from CIHR. These accomplishments, all achieved after receiving eHIPP funding, collectively represent CAD$ 16 million in CIHR funding and CAD$ 12.7 million in partner investments, encompassing both cash and in-kind contributions.

### Advance knowledge

#### eHealth interventions and research outputs

The research teams reported 36 health solutions that were co-designed and researched. The solutions included virtual care/telemedicine (*n* = 10 solutions); home health monitoring applications (*n* = 9), patient portals/web-based self-management tools (*n* = 6) or patient wearables/sensors (*n* = 4). Less frequent solutions included virtual dashboards (*n* = 3), e-referrals/e-consults (*n* = 2) and system reporting applications (*n* = 2). The main beneficiaries of the interventions were reported to be patients (90%) and/or providers (48%). Reported target populations included caregivers, racialized groups and/or underserved communities, university students, health policymakers or people with more specific clinical phenotypes (surgical or oncology patients, children with neurodevelopmental disorders, people with cardiometabolic disease who require remote patient monitoring). Final reports indicate that research findings from the eHIPP interventions have been disseminated through various channels, including 79 peer reviewed publications, 194 presentations, 38 media interviews or articles and 1 book chapter.

In addition to developing and implementing digital health interventions, survey respondents and interviewees reported other outcomes of the research, such as enhanced understanding of population needs and contexts, plans for commercialization and informing policy, advances in methodology, academic contributions through publications and presentations and infrastructure development in the form of a new research lab and a start-up firm.

#### The value of partnerships

The eHIPP program was designed with an emphasis on fostering partnerships and the co-design of innovations – both key impact enablers, per the CHSPRA framework. Respondents indicated that slightly more than half of their partnerships were preexisting (55%), whilst several partnerships, particularly with patient partners, were developed for the eHIPP program. Survey respondents and interviewees recognized that relationship-building takes time. When questioned about sustainability of their partnerships, the majority of survey respondents reported that their partnerships would be at least somewhat sustained beyond a 5-year period. Specifically, 95% of respondents felt that their relationship with health providers would endure beyond this 5-year time frame, and similar levels of sustainability were reported for partnerships with other stakeholders (i.e. 90% with patient partners, 82% with industry representatives and 77% decision-makers).

The survey responses and interview evidence corroborated that the meaningful engagement of partners played a positive and pivotal role in the research projects. When asked whether partnerships were crucial to the success of the research, respondents reported that they strongly valued their partnerships with healthcare providers (95%), industry (91%), patients (86%) and policy/decision-makers (52%). Other types of reported partnerships included engagement with Indigenous communities, the not-for-profit sector and government and academic institutions.

**Fig. 2:**
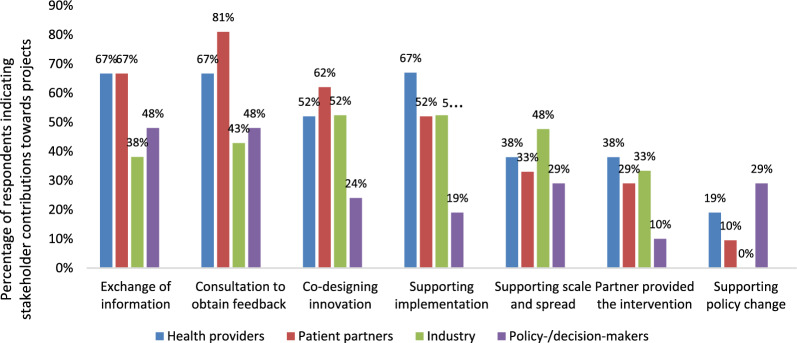
eHIPP partner contributions to research as reported by survey respondents

Researchers were asked to indicate the nature and contributions of their partnerships to the various research and development activities (Fig. 2). Results indicate that industry partnership primarily supported implementation, co-design and scale and spread of innovations. Both survey responses and interviews affirmed that patient representatives were most frequently engaged for input/feedback, co-design and support for implementation of the eHealth innovation. Some researchers attributed the successes of their interventions to the contributions of their patient representatives, with one researcher stating: “All through this journey of course, we need the health policymakers, we need the health professionals, we need the researchers. But what anchors them very well is that we have patients all along the way as patient partners with lived experiences to guide us”. Patient representatives also expressed feeling genuinely involved in the co-design process, with one reporting: “I really felt like we were partners”.

Health providers mainly contributed to supporting implementation, consultations for feedback and information exchange. Amongst all partner groups, health providers were perceived to offer the most substantial support for implementation given their expertise in health-related issues and their deep understanding of the needs and preferences of target populations. Policy/decision-makers provided feedback and consultation and exchange of information, as well as supported policy change and scale and spread and were less involved in the implementation process.

Interviewees discussed the challenges associated with multi-stakeholder partnerships. The main challenge reported by researchers were the shifting priorities of the industry partners and policy/decision-makers over the lifecycle of the grant resulting in the de-prioritization of research projects. Policy/decision-makers also noted the misalignment of the eHealth work with the evolving needs and structure of the healthcare system. One policymaker highlighted the discordance between research and policy timelines, stating that the development and implementation of research interventions is too long and careful consideration is required to ensure research addresses future needs of the health system.

Overall, partnerships were perceived as key to success of the projects, with 62% of survey respondents indicating that their project’s impact would not have been possible without the many partnerships developed throughout the grant cycle. This was further emphasized during an interview with one researcher, who stated: “You cannot have impacts without all of those people”.

### Informing decisions, policy or practice

Survey respondents were asked the extent to which their research influenced practice or policy, with 52% indicating that their project influenced health system practices and 33% reporting their project informed health policy. The research teams published a total of seven reports/policy briefs/clinical guidelines, whilst others reported informing policy and practice by engaging with government entities, either by participating in provincial committees or meetings. These interactions provided them an opportunity to explore how their solutions could lead to system change. Some researchers noted the turnover of their health provider and decision/policymaker partners as hindering or delaying potential practice- and policy-related impacts. However, decision/policymakers recognized the value of this work to improve health system performance and provider experience, with one stating: “So, if you think about clinicians, there must be so many opportunities for how to better use technology to influence everything from a discrete clinical decision to how they choose to prioritize whatever set of patients they’re going to see that day” (Policymaker).

### Enhance health and health system outcomes

Survey respondents were asked to indicate the current stage of their innovation funded by the eHIPP program. The majority reported that their innovations were either being implemented, spread to other settings/contexts/populations or scaled to expand the coverage of their solution (76%), whilst others reporting they were in the development/testing/piloting stages. Amongst those who were spreading and scaling their solutions, 31% reported spreading their solution within their organization and 81% reported spreading and scaling beyond their organization within their province/territory and/or to other regions across Canada. Additionally, 19% reported that their intervention was spread and/or scaled internationally.

**Fig. 3:**
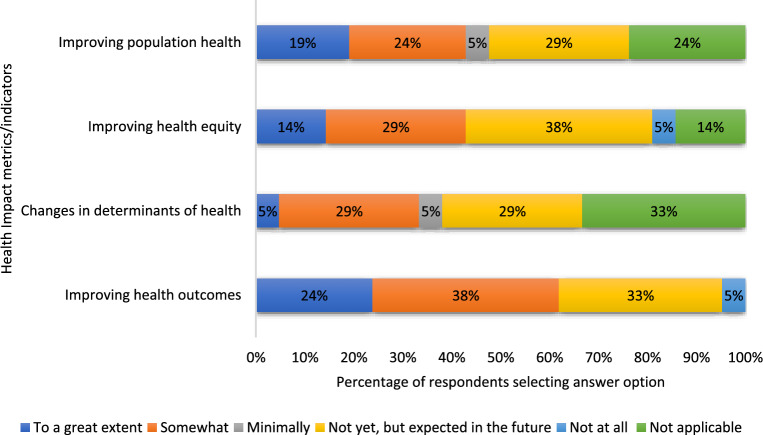
Extent to which eHIPP solutions contributed to health impacts

**Fig. 4:**
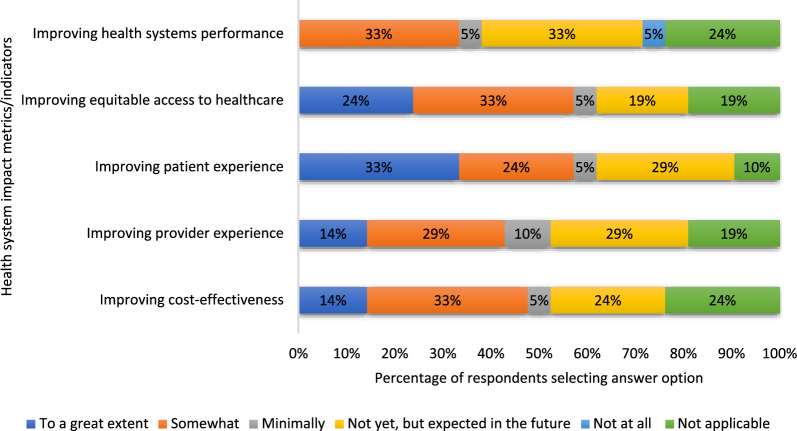
Extent to which eHIPP solutions contributed to health system impacts

When asked about perceived health-related impacts (Fig. 3), the respondents reported that their research contributed to enhanced health outcomes (62%), improved population health (48%), advances in health equity (43%) and changes in determinants of health (38%) for the target populations studied. With respect to the health system impacts of their innovation (Fig. 4), the most substantial impact of the research was on enhanced patient experience (62%) and improving equitable access to healthcare (62%). Slightly more than half of the respondents (52%) indicated that their research improved cost-effectiveness and provider experience, whilst enhancements in health system performance (38%) were less frequently reported.

Interviewees expressed how difficult it can be to assess health and health system impacts. One researcher said: “We’ve laid the groundwork for improving health outcomes. But we don’t know if we have improved health outcomes, because it was an overly ambitious timeframe for evaluation, for something that involves such radical change”.

### Enablers and barriers to impact

The market demand for digital health interventions, along with their co-creation with patient partners, health providers and policy/decision-makers, were identified as significant enabling factors for achieving impact. The national reputation and credibility of the eHIPP program was also perceived as an enabler as it helped to pave way for partnerships that meaningfully strengthened the research. The coronavirus disease 2019 (COVID-19) pandemic’s negative impacts on access to health services acted as a catalyst for innovation, fostering more market demand and favourable attitudes towards digital health interventions and accelerating the shift from face-to-face health services to digital alternatives. One researcher shared their experience: “We were having a hard time getting in there, like being accepted as a legitimate intervention approach, right? … And then COVID hits, and everyone has to use this [digital health intervention] and realizes: Hey, it can work”. However, the pandemic also presented challenges as it complicated participant recruitment and intervention testing. The pandemic also affected the time of health providers and policy/decision-makers had to support research and stimulated high turnover rates of policy/decision-makers and health workers due to burnout.

## Discussion

With the digital transformation of health systems on the rise [2, 25], there is a critical need to understand the effectiveness and impacts of digital health solutions. This includes assessing their potential benefits, costs, long-term sustainability and implications on health equity, as well as understanding the effects of research investments intended to enhance capacity in this area. This paper explores the research impacts of the eHIPP program through the lens of the CAHS and CHSPRA frameworks. This study represents the first application of these frameworks to assess the research impact of a large-scale pan-Canadian digital health investment.

The eHIPP program developed capacity by harnessing investments from CIHR, industry and health system partners, promoting intersectoral partnerships, funding large pan-Canadian research teams and designing and integrating digital health innovations within the healthcare system. The program was designed to encourage conditions of success, specifically in terms of intersectoral partnerships and the required 1:1 matching of CIHR’s investment, ensuring a commitment from partnering organizations. Together, CIHR and partners invested CAD$ 42.6 million, resulting in 22 funded research teams who were supported by 181 unique organizations/institutions across Canada.

The eHIPP program accelerated both the overall professional growth of lead investigators and their research growth in the area of digital health, as mentioned in interviews and quantified in survey and final report data. The data revealed that 127 trainees were supervised throughout the grant lifecycle, research evidence was disseminated through 312 knowledge products (publications, presentations and media outputs), and the majority of lead investigators received subsequent CIHR grants (either as lead or co-applicants).

Strong partnerships and co-design approaches were also key funding design elements of the eHIPP program that facilitated successful progress of the research projects. The nature and importance of the partnerships were highly valued by respondents and interviewees, with many reporting that real-world impact would not have been possible without the collaboration of all partners involved in the research. Co-design principles help to guarantee that innovations are not only relevant to and feasible for patients and providers, but also that they have the potential for long-term viability and equitable integration into the health delivery system. Many co-design frameworks exist [26–28], including a set of principles developed by Voorheis et al. in 2024 tailored specifically for co-designing equitable digital health solutions [29]. The Principles for Equitable and Inclusive Digital Health Co-Design framework outlines the necessary actions to follow across all phases of co-design when engaging with patient partners and communities, especially those from equity-deserving groups. This framework may serve as an important resource for future digital health research and implementation.

Mobilizing evidence into policy and practice was a key objective of this digital health research program, but difficult to directly attribute to research investments. In this study, half of respondents reported that the research influenced healthcare practice and one-third reported that it informed health policy decisions, which is challenging for researchers and funders to assert with confidence. For instance, the Public Health Agency of Canada led a study on another program in 2021, which found that providing evaluation support to teams enhanced their ability to understand the influence their projects were having on practice and policy [30]. Additionally, the evaluation expertise improved the teams’ capacity to effectively mobilize their evidence for policy and system transformation, with 90% of projects reporting having long-term influence on policy and practice.

The Quintuple Aim is recognized as a key set of principles for driving improvements in health systems and is progressively being adopted in Canada as a framework for evaluating health system performance [22, 23]. Using an expansive view of the Quintuple Aim goals as indicators, the majority of projects were reported to improve health outcomes, enhance patient experience and increase equitable access to care, whilst half of the projects were cost-effective, improved provider wellbeing and enhanced overall population health.

There is increasing pressure for research funders to show accountability to the public for their investments and showcase their real-world impacts [31–34], but the emerging field of RIA comes with many challenges. Some of these challenges include the need to adapt current impact frameworks to meet the needs of the program under evaluation [32, 34]; the time-consuming, resource-intensive and complex nature of assessing impact within organizations who are juggling competing demands resulting in the need to commission external support [33, 34]; and the imperative to develop new tools that align with the impact framework being used to capture impacts in a systematic way [31–33]. This is further compounded by the challenges of determining attribution of outcomes to the funding investment; the length of time it takes to realize broader health, societal and economic impacts; and the uncertainty of the best timing to assess research impact [8, 32]. Additionally, to assess impact, research funders rely heavily on information provided by the lead investigators, who are believed to under-report the extent to which their research had influence, as has been observed in previous studies [33, 34], and whose perspective may not adequately capture or represent the perspectives of others (e.g. policymakers, patients) on the team.

Despite the challenges, many organizations are prioritizing research impact and RIAs, recognizing the clear value in understanding the degree to which tax-payer dollars are advancing health outcomes and system performance [7, 8, 35]. The CIHR 2021–2031 Strategic Plan [36] is centred on the notion that the Canadian health research ecosystem will “…be internationally recognized as inclusive, collaborative, transparent, culturally safe, and focused on real world impact”. CIHR’s newly developed Research Excellence Framework further highlights the notion that research must be inclusive, conducted respectfully and consider the diversity of all perspectives to ensure maximal broader impact [37]. CIHR has taken several steps to advance its vision of funding research that has broad real-world impact, including establishing a new Impact Assessment Unit, signing on to the San Francisco Declaration on Research Assessment (DORA) [38] and building resources for researchers [39] and peer reviewers [40] on how to highlight and assess research impact, respectively. Furthermore, CIHR’s Institutes are developing impact assessment infographics [14–16] on funded programs, creating impact spotlights [41, 42] and casebooks that highlight impact narratives [43] or evidence briefs [44] to maximize the dissemination of knowledge at the end of the grant funding stage.

To address the complexities involved in understanding and assessing research impact, as well as in mobilizing evidence into policy and practice, it is essential for funders, researchers, policymakers and other relevant stakeholders to collaborate and learn from one another. By working together, these groups can enhance their collective understanding and support the integration of research findings into policy and practice. The Research on Research Institute [45], the Transforming Evidence Funders Network [46] and CHSPRA [12] are examples of networks that have come together as a collective with diverse perspectives to address some of challenges related to research impact including how to set research priorities, understanding how evidence is used and how to effectively assess research impact. As research funders increasingly evaluate and assess their programs and investments, these collaborative efforts will help us to continually learn, iterate and improve the processes involved in assessing research impact. 

## Study limitations

Whilst the authors sought the perspectives of partners (providers, decision/policymakers, industry representatives and patients) regarding the impacts of eHIPP, it was challenging to source their insights. Due to CIHR’s privacy policies to safeguard the contact information of partners, direct contact with partners was not feasible. The study team attempted to rely on the lead investigators to forward survey requests to partners; however, this approach proved challenging, particularly with a study focussed on decision-making and health system impacts. Out of the 21 survey responses received, 12 were from lead investigators who were identifiable, whilst the remaining 9 responses were unidentifiable, presumably submitted from partners. To counterbalance and glean other perspectives, over half the interviews were conducted with partners who were not researchers. Others have also identified the challenging yet important nature of gaining insights from research partners when assessing impact [8, 17, 47].

Lead investigators are subject to several conditions of funding when awarded a grant. At the time of application, the submission of a final report was mandated as a condition for receiving funding, whilst completing the impact survey was not. As of writing this manuscript, only 17 final reports have been submitted and were analysed for the assessment. Moving forward, funding agencies will need to establish and implement clear guidelines for collecting impact data, which are known to applicants at the time of grant application. This will help to ensure effective evaluation of future large-scale research programs.

## Conclusions

In this paper, the authors explore and assess the key outcomes and research impacts of the eHIPP program, using the CAHS and CHSPRA frameworks. To the best of our knowledge, this is the first study to apply these frameworks to assess the impact of a major pan-Canadian investment in digital health research. The findings reveal that collaboration amongst diverse stakeholders, strong partnerships and co-design approaches are highly valued and instrumental for implementing patient-centred, cost-effective digital health solutions. These strategies effectively address the needs of populations they aim to serve, improve patient and provider experience, enhance health equity and equitable access to care and influence policy and practice. Furthermore, this study advances the science of RIA and underscores the critical role of RIAs as an essential tool for research funders to grasp the long-term effects and impacts of their programs and the value of their investments.

## Supplementary Information

Additional file 1. CIHR Final Report Template. The CIHR final report template that the eHIPP lead investigators were required to complete.

Additional file 2. eHealth Innovations Partnership Program – Impact Analysis Survey. The online survey sent to the eHIPP lead investigators and their project partners to compete.

Additional file 3. Information about the eHIPP research teams and projects. A list of the funded eHIPP projects. The table includes the key eHIPP priority area addressed, project title, and the province of the eHIPP lead investigator.

Additional file 4. eHealth Innovations Partnership Program – Impact Analysis Interview Guide. The eHIPP impact analysis interview guide that guided the discussion with the 13 interview participants.

## Data Availability

No datasets were generated or analysed during the current study.

## Abbreviations

CAHS: Canadian Academy of Heath Science
CHSPRA: Canadian Health Services and Policy Research Alliance
CIHR: Canadian Institutes of Health Research
eHIPP: eHealth Innovations Partnership Program
DORA: Declaration on Research Assessment
HSPR: Health services and policy research
IA: Institute of Aging
IHDCYH: Institute of Human Development, Child and Youth Health
IHSPR: Institute of Health Services and Policy Research
INMHA: Institute of Neuroscience, Mental Health and Addictions
KM: Knowledge mobilization
RIA: Research impact assessment
