# Supplementary material for: Research impact assessment of a Canadian digital health funding program: a case study
Source: Health Res Policy Syst. 2025 Jun 23;23:81. doi: 10.1186/s12961-025-01356-2 (PMC12183889; doi:10.1186/s12961-025-01356-2)
Supplement: Supplementary file 1 — Additional file 1. CIHR Final Report Template. The CIHR final report template that the eHIPP lead investigators were required to complete. [file 12961_2025_1356_MOESM1_ESM.pdf]

Canadian Institutes of Health Research | Instituts de recherche en santé du Canada

PROTECTED WHEN COMPLETED

Appl. #
FRN #

Final Report

Update Profile and Grant Information - NPI Profile

Nominated Principal Applicant/Candidate

Surname | Given Names

Mailing Address:

Telephone | Fax

How long have you been an independent researcher? _____

Are you a clinician, health practitioner, health professional, or health provider who is in a role in which you make clinical judgements and/or decisions? ○ Yes ○ No

SAMPLE

Update Profile and Grant Information - Research Team

Other Applicants

Name	Role
Institution	
Name	Role
Institution	
Name	Role
Institution	
Name	Role
Institution	
Name	Role
Institution	
Name	Role
Institution	
Name	Role
Institution	
Name	Role
Institution	
Name	Role
Institution	
Name	Role
Institution	
Name	Role
Institution	
Name	Role
Institution	
Name	Role
Institution	
Name	Role
Institution	
Name	Role
Institution	
Name	Role
Institution	
Name	Role
Institution	
Name	Role
Institution	

SAMPLE

Canadian Institutes of Health Research | Instituts de recherche en santé du Canada

Update Profile and Grant Information - Financial Support

Enter the amount of the contribution received in financial support from the following organizations:

**Organization**	Yes - Cash support
CIHR Funding	$ ___________
Academia	$ ___________
Academia	$ ___________
Academia	$ ___________
Academia	$ ___________
Academia	$ ___________
Academia	$ ___________
Academia	$ ___________
Academia	$ ___________
Academia	$ ___________
Academia	$ ___________
International	$ ___________
International	$ ___________
International	$ ___________
International	$ ___________
International	$ ___________
International	$ ___________
International	$ ___________
International	$ ___________
International	$ ___________
International	$ ___________
Private	$ ___________
Private	$ ___________

SAMPLE

Canadian Institutes of Health Research | Instituts de recherche en santé du Canada

Update Profile and Grant Information - Financial Support

Enter the amount of the contribution received in financial support from the following organizations:

**Organization**		Yes – Cash support
Private		$ ___________
Private		$ ___________
Private		$ ___________
Public		$ ___________
Public		$ ___________
Public		$ ___________
Public		$ ___________
Public		$ ___________
Voluntary		$ ___________
Voluntary		$ ___________
Voluntary		$ ___________
Voluntary		$ ___________
Voluntary		$ ___________
	**Total**	$ ___________

SAMPLE

Canadian Institutes of Health Research | Instituts de recherche en santé du Canada

Research and Knowledge Translation Practices - Stakeholders

Were any of the stakeholders in the list below involved in the research process? If YES, how?

**Project Step** / **Type of Stakeholder**	INVOLVED	Development of the research idea / question	Development of the protocol	Data collection phase/project implementation	Interpretation of the results	End of Grant KT activities
Health System/Care Practitioners/Public Health Practitioners	☐	☐	☐	☐	☐	☐
Patients/Consumers of Health System/Care	☐	☐	☐	☐	☐	☐
Study Stakeholders (who are formally listed in the grant application)	☐	☐	☐	☐	☐	☐
Health System/Care Managers	☐	☐	☐	☐	☐	☐
Health System/Care Professional Organizations	☐	☐	☐	☐	☐	☐
Federal/Provincial Representatives	☐	☐	☐	☐	☐	☐
Community/Municipal Organizations	☐	☐	☐	☐	☐	☐
Consumer Groups/Charitable Organizations	☐	☐	☐	☐	☐	☐
Industry	☐	☐	☐	☐	☐	☐
The media	☐	☐	☐	☐	☐	☐
Other Researchers/Academics (excluding Study Stakeholders)	☐	☐	☐	☐	☐	☐
Other	☐	☐	☐	☐	☐	☐
Other	☐	☐	☐	☐	☐	☐
Other	☐	☐	☐	☐	☐	☐
Other	☐	☐	☐	☐	☐	☐
Other	☐	☐	☐	☐	☐	☐
Other	☐	☐	☐	☐	☐	☐
Other	☐	☐	☐	☐	☐	☐
Other	☐	☐	☐	☐	☐	☐
Other	☐	☐	☐	☐	☐	☐
Other	☐	☐	☐	☐	☐	☐

SAMPLE

Canadian Institutes of Health Research | Instituts de recherche en santé du Canada

**Research Findings – Lay Summary and Implications of Key Findings**

SAMPLE

Research Findings - Contribution to CIHR mandate

To what extent do you feel the research results from this grant contributed to the CIHR mandate?

	Not at all	Some extent	Great extent	May in the future
**1.** Creating new health knowledge	◯	◯	◯	◯
**2.** Translating the knowledge from the research setting into real world applications	◯	◯	◯	◯
**3.** Improving health for Canadians	◯	◯	◯	◯
**4.** Creating more effective health services and products	◯	◯	◯	◯
**5.** Strengthening the Canadian health care system	◯	◯	◯	◯

SAMPLE

Canadian Institutes of Health Research | Instituts de recherche en santé du Canada

Research Findings - Groups aware of findings

Which groups are already/need to be aware of your findings?

Groups	Need to be aware		Are already aware		
	Yes	No	Yes	No	Don't know
Health System/Care Practitioners/Public Health Practitioners					
Patients/Consumers of Health Care System/Care					
Study Stakeholders (who are formally listed in the grant application)					
Health System/Care Managers					
Health System/Care Professional Organizations					
Federal/Provincial Representatives					
Community/Municipal Organizations					
Consumer Groups/Charitable Organizations					
Industry					
The media					
Other Researchers/Academics (excluding Study Stakeholders)					
Other					
Other					
Other					
Other					
Other					
Other					
Other					
Other					
Other					
Other					

SAMPLE

Canadian Institutes of Health Research | Instituts de recherche en santé du Canada

Broader Impacts - Human Research Participants

How many human research participants were enrolled in this study?

○ No human research participants enrolled

○ If yes, total number of human research participants:

Participant Types	Number
Males	
Females	
Not Collected	
Total	

How many institutions were involved?

How many institutions?	From which countries?	From which province? (Canada only)

Have human research participants benefited as a result of participating in this grant?

○ No benefit to human research participants

○ Yes, please describe

Describe (max. 1000 characters including spaces)

SAMPLE

Broader Impacts - Stakeholders

To what extent and how has your grant had an impact on the following stakeholders?

	Influenced						How
**Stakeholder(s)**	**N/A**	**Don't know**	**Not at all**	**A little extent**	**Some extent**	**Great extent**	
Health System/Care Practitioners/Public Health Practitioners							
Patients/Consumer of Health System/Care							
Study Stakeholders (who are formally listed in the grant application)							
Health System/Care Managers							
Health System/Care Professional Organizations							
Federal/Provincial Representatives							
Community/Municipal Organizations							
Consumer Groups/Charitable Organizations							

SAMPLE

Industry	◯	◯	◯	◯	◯	◯	
The media	◯	◯	◯	◯	◯	◯	
Researchers/Academics (excluding Study Stakeholders)	◯	◯	◯	◯	◯	◯	
Research Funding Organizations	◯	◯	◯	◯	◯	◯	
Other	◯	◯	◯	◯	◯	◯	
Other	◯	◯	◯	◯	◯	◯	
Other	◯	◯	◯	◯	◯	◯	
Other	◯	◯	◯	◯	◯	◯	
Other	◯	◯	◯	◯	◯	◯	

SAMPLE

Canadian Institutes of Health Research | Instituts de recherche en santé du Canada

Broader Impacts - Outcomes

Which of the following have resulted or will result from this grant?

	Advanced		Newly developed		May in the future	Please describe with an example
Outcomes	Yes	No	Yes	No		
Research method	○	○	○	○	○	
Theory	○	○	○	○	○	
Replication of research findings	○	○	○	○	○	
Tool, technique, instrument, or procedure	○	○	○	○	○	
Professional practice	○	○	○	○	○	
Policies, guidelines or programs	○	○	○	○	○	
Information or guidance for patients or public	○	○	○	○	○	
Patients' or public behaviour(s)	○	○	○	○	○	

SAMPLE

Vaccines/Drugs						
Software/Database						
Patent (filled or obtained)						
Product licence						
Spin-off company						
Intellectual property claim						
Direct cost savings (individual, organization, system, or population level)						
Findings cited by others (e.g. finding referenced/included in subsequent synthesis, practice guideline, etc.						

SAMPLE

Canadian Institutes of Health Research | Instituts de recherche en santé du Canada

**Broader Impacts - Impact/Contribution of findings** (max. 2000 characters)
**Is there anything else CIHR should know about how findings from this grant may be having an impact/make an important contribution?** ○ **Yes** ○ **No**

SAMPLE

Canadian Institutes of Health Research
Instituts de recherche en santé du Canada

Research Capacity and Training - List of all staff

Please list all staff including trainees involved in this current grant (both paid and not paid).

Type		Unique Number of Individuals Paid from Grant	Total # FTE Paid from Grant	Unique Number of Individuals involved but NOT Paid	Total # FTE NOT Paid from Grant
Researcher					
Research Assistant					
Research Technician					
Trainees	Postdoctoral (post Ph.D) fellows				
	Post health professional degree (MD, BScN, DDS, etc.)				
	Ph.D. students				
	Fellows not pursuing a Master's or Ph.D Doctoral				
	Master's students				
	Under graduate student				
Other					
Other					
Other					
Other					
Other					
Other					
Other					
Other					
Other					
Other					

SAMPLE

Research Capacity and Training - Attracting new researchers

CIHR is interested in whether Canada is attracting new health researchers to Canada to build capacity. Did your grant attract foreign researchers, research staff or trainees? ○ Yes ○ No

Type	From which countries?	How many individuals?

SAMPLE

Canadian Institutes of Health Research | Instituts de recherche en santé du Canada

Research Capacity and Training - Qualifications for members

Has participation in this grant led to formal qualifications (e.g. PhD) for any members of the project team or is it likely to do so? ○ Yes ○ No

Qualifications	Year degree awarded or expected	Contributions from specific project			
		A little extent	Some extent	Considerable extent	Great extent
		○	○	○	○
		○	○	○	○
		○	○	○	○
		○	○	○	○
		○	○	○	○
		○	○	○	○
		○	○	○	○
		○	○	○	○
		○	○	○	○
		○	○	○	○

Advancing Knowledge – Scientific Production

Please indicate the number of each of the following items related to this grant, by you or others on your team

	# Published	# Submitted
Journal Articles		
Books/Book chapters		
Reports/Technical reports		

	# Invited	# Others
Presentations		

	In Canada			International		
	#Print	#Broadcast	#Internet	#Print	#Broadcast	#Internet
Interviews with Journalists/Articles in Mass Media						

SAMPLE

Canadian Institutes of Health Research | Instituts de recherche en santé du Canada

Advancing Knowledge - Open access publications

Have you adhered to the following requirements outlined in the CIHR Open Access Policy?

Responsibilities	Yes	No	N/A
Ensure that all research papers generated from CIHR funded projects are freely accessible through the Publisher's website or an online repository within twelve months of publication	○	○	○
Deposit bioinformatics, atomic, and molecular coordinate data into the appropriate public database (e.g. gene sequences deposited in GenBank) immediately upon publication of research results	○	○	○
Retain original data sets for a minimum of five years (or longer if other policies apply)	○	○	○
Acknowledge CIHR support by quoting the funding reference number in journal publications	○	○	○

If you selected no, please explain why you were not able to comply: (max. 2000 characters including spaces)

○ I was funded prior to January 1st 2008

○ Other, please explain for each responsibility:

SAMPLE

Canadian Institutes of Health Research | Instituts de recherche en santé du Canada

Advancing Knowledge - Peer-reviewed publications

Information of Peer-Reviewed Publications

1	Title*	Supported by this CIHR grant ○ Fully ○ Partially<br>Volume No.
	Journal*	Number
	Primary Author Name*	Page No.
	Author(s)	Year
	URL	
	DOI	
2	Title*	Supported by this CIHR grant ○ Fully ○ Partially<br>Volume No.
	Journal*	Number
	Primary Author Name*	Page No.
	Author(s)	Year
	URL	
	DOI	
3	Title*	Supported by this CIHR grant ○ Fully ○ Partially<br>Volume No.
	Journal*	Number
	Primary Author Name*	Page No.
	Author(s)	Year
	URL	
	DOI	
4	Title*	Supported by this CIHR grant ○ Fully ○ Partially<br>Volume No.
	Journal*	Number
	Primary Author Name*	Page No.
	Author(s)	Year
	URL	
	DOI	
5	Title*	Supported by this CIHR grant ○ Fully ○ Partially<br>Volume No.
	Journal*	Number
	Primary Author Name*	Page No.
	Author(s)	Year
	URL	
	DOI	

SAMPLE

<table>
<tr><td rowspan="7">6</td><td>Title*</td><td>Supported by this CIHR grant
○ Fully ○ Partially</td></tr>
<tr><td></td><td>Volume No.</td></tr>
<tr><td>Journal*</td><td>Number</td></tr>
<tr><td>Primary Author Name*</td><td>Page No.</td></tr>
<tr><td>Author(s)</td><td>Year</td></tr>
<tr><td colspan="2">URL</td></tr>
<tr><td colspan="2">DOI</td></tr>
<tr><td rowspan="7">7</td><td>Title*</td><td>Supported by this CIHR grant
○ Fully ○ Partially</td></tr>
<tr><td></td><td>Volume No.</td></tr>
<tr><td>Journal*</td><td>Number</td></tr>
<tr><td>Primary Author Name*</td><td>Page No.</td></tr>
<tr><td>Author(s)</td><td>Year</td></tr>
<tr><td colspan="2">URL</td></tr>
<tr><td colspan="2">DOI</td></tr>
<tr><td rowspan="7">8</td><td>Title*</td><td>Supported by this CIHR grant
○ Fully ○ Partially</td></tr>
<tr><td></td><td>Volume No.</td></tr>
<tr><td>Journal*</td><td>Number</td></tr>
<tr><td>Primary Author Name*</td><td>Page No.</td></tr>
<tr><td>Author(s)</td><td>Year</td></tr>
<tr><td colspan="2">URL</td></tr>
<tr><td colspan="2">DOI</td></tr>
<tr><td rowspan="7">9</td><td>Title*</td><td>Supported by this CIHR grant
○ Fully ○ Partially</td></tr>
<tr><td></td><td>Volume No.</td></tr>
<tr><td>Journal*</td><td>Number</td></tr>
<tr><td>Primary Author Name*</td><td>Page No.</td></tr>
<tr><td>Author(s)</td><td>Year</td></tr>
<tr><td colspan="2">URL</td></tr>
<tr><td colspan="2">DOI</td></tr>
<tr><td rowspan="7">10</td><td>Title*</td><td>Supported by this CIHR grant
○ Fully ○ Partially</td></tr>
<tr><td></td><td>Volume No.</td></tr>
<tr><td>Journal*</td><td>Number</td></tr>
<tr><td>Primary Author Name*</td><td>Page No.</td></tr>
<tr><td>Author(s)</td><td>Year</td></tr>
<tr><td colspan="2">URL</td></tr>
<tr><td colspan="2">DOI</td></tr>
</table>

SAMPLE

Canadian Institutes of Health Research | Instituts de recherche en santé du Canada

11	Title*	Supported by this CIHR grant ◯ Fully ◯ Partially
		Volume No.
	Journal*	Number
	Primary Author Name*	Page No.
	Author(s)	Year
	URL	
	DOI	
12	Title*	Supported by this CIHR grant ◯ Fully ◯ Partially
		Volume No.
	Journal*	Number
	Primary Author Name*	Page No.
	Author(s)	Year
	URL	
	DOI	
13	Title*	Supported by this CIHR grant ◯ Fully ◯ Partially
		Volume No.
	Journal*	Number
	Primary Author Name*	Page No.
	Author(s)	Year
	URL	
	DOI	
14	Title*	Supported by this CIHR grant ◯ Fully ◯ Partially
		Volume No.
	Journal*	Number
	Primary Author Name*	Page No.
	Author(s)	Year
	URL	
	DOI	
15	Title*	Supported by this CIHR grant ◯ Fully ◯ Partially
		Volume No.
	Journal*	Number
	Primary Author Name*	Page No.
	Author(s)	Year
	URL	
	DOI	

SAMPLE

Information of Presentations		
1	Title*	☐ This was an invited presentation
	Author(s)	
	Type of presentation	
	Workshop / Conference Name	Date
	Location	
	URL	
2		☐ This was an invited presentation
	Author(s)	
	Type of presentation	
	Workshop / Conference Name	Date
	Location	
	URL	
3	Title*	☐ This was an invited presentation
	Author(s)	
	Type of presentation	
	Workshop / Conference Name	Date
	Location	
	URL	
4	Title*	☐ This was an invited presentation
	Author(s)	
	Type of presentation	
	Workshop / Conference Name	Date
	Location	
	URL	
5	Title*	☐ This was an invited presentation
	Author(s)	
	Type of presentation	
	Workshop / Conference Name	Date
	Location	
	URL	

SAMPLE

6	Title*	☐ This was an invited presentation
	Author(s)	
	Type of presentation	
	Workshop / Conference Name	Date
	Location	
	URL	
7	Title*	☐ This was an invited presentation
	Author(s)	
	Type of presentation	
	Workshop / Conference Name	Date
	Location	
	URL	
8	Title*	☐ This was an invited presentation
	Author(s)	
	Type of presentation	
	Workshop / Conference Name	Date
	Location	
	URL	
9	Title*	☐ This was an invited presentation
	Author(s)	
	Type of presentation	
	Workshop / Conference Name	Date
	Location	
	URL	
10	Title*	☐ This was an invited presentation
	Author(s)	
	Type of presentation	
	Workshop / Conference Name	Date
	Location	
	URL	

SAMPLE

Canadian Institutes of Health Research | Instituts de recherche en santé du Canada

11	Title*		☐ This was an invited presentation
	Author(s)		
	Type of presentation		
	Workshop / Conference Name		Date
	Location		
	URL		
12	Title*		☐ This was an invited presentation
	Author(s)		
	Type of presentation		
	Workshop / Conference Name		Date
	Location		
	URL		
13	Title*		☐ This was an invited presentation
	Author(s)		
	Type of presentation		
	Workshop / Conference Name		Date
	Location		
	URL		
14	Title*		☐ This was an invited presentation
	Author(s)		
	Type of presentation		
	Workshop / Conference Name		Date
	Location		
	URL		
15	Title*		☐ This was an invited presentation
	Author(s)		
	Type of presentation		
	Workshop / Conference Name		Date
	Location		
	URL		

SAMPLE
