# Supplementary material for: Research impact assessment of a Canadian digital health funding program: a case study
Source: Health Res Policy Syst. 2025 Jun 23;23:81. doi: 10.1186/s12961-025-01356-2 (PMC12183889; doi:10.1186/s12961-025-01356-2)
Supplement: Supplementary file 2 — Additional file 2. eHealth Innovations Partnership Program – Impact Analysis Survey. The online survey sent to the eHIPP lead investigators and their project partners to compete. [file 12961_2025_1356_MOESM2_ESM.pdf]

eHealth Innovations Partnership Program – Impact Analysis Survey

Background

1. Which eHealth grant supported your work? (If you have received more than one grant type, please choose all that apply).

☐ Catalyst Grants
☐ eHealth Innovations Partnership Program (eHIPP)
☐Spread and Scale of Community-Based Primary Health Care and eHealth Innovations
☐ Active and Assisted Living (AAL) Program

2. What kind of ehealth innovation have you been working on?
☐Virtual care / telemedicine
☐e-referral/e-consult
☐Patient portal
☐Wearable/sensor
☐Home health monitoring application
☐Virtual dashboard
☐Other: ______________________

3. What is the current stage of your innovation that was funded through the program?
☐In development / testing / piloting
☐Under evaluation
☐Implementing
☐Scaling up
☐Spreading to other settings/contexts/populations

Outcomes and Impacts

4. What have been the outcomes of this eHIPP supported work?

5. Have your fundings/evidence indicated that your eHIPP project contributed to the following health impacts?

	To a great deal	Somewhat	Minimally	Not at all	Not yet, but expected in the future	Not applicable
Improving health outcomes	◯	◯	◯	◯	◯	◯
Changes in determinants of health	◯	◯	◯	◯	◯	◯
Improving health equity	◯	◯	◯	◯	◯	◯
Improving population health	◯	◯	◯	◯	◯	◯
Other health impacts	◯	◯	◯	◯	◯	◯

Please specify other health impacts

[ ]

6. Have your findings/evidence indicated that your eHII project contributed to the following health system impacts

	To a great deal	Somewhat	Minimally	Not at all	Not yet, but is expected in the future	Not applicable
Improving cost-effectiveness	○	○	○	○	○	○
Improving provider experience	○	○	○	○	○	○
Improving patient experience	○	○	○	○	○	○
Improving equitable access to healthcare	○	○	○	○	○	○
Changes in health systems practice(s)	○	○	○	○	○	○
Changes in health policy	○	○	○	○	○	○
Improving health systems performance	○	○	○	○	○	○
Other health systems impacts	○	○	○	○	○	○

Please specify other health system/policy impacts

[ ]

7. Who are the main intended beneficiaries of your eHIPP work? Please choose all that apply.
   ☐Patients
   ☐Providers
   ☐Specific populations: ____________________

8. Has your eHealth innovation spread or been scaled up?
   ☐Within your organization
   ☐Within your municipality
   ☐Within the province/territory
   ☐To other provinces/territories
   ☐Internationally

9. Did eHIPP in any way prepare you for working on solutions to address the COVID pandemic? If so, please elaborate.

10. Do you think that your eHIPP work had unintended negative impacts
    ☐I don't know
    ☐No
    ☐Yes. If yes, please elaborate: ________________________

11. Have there been any broader economic and societal impacts of your eHII work? If so, please elaborate.

12. Has your eHIPP work included partnerships with? Please select all that apply.
   ☐Industry
   ☐Healthcare professionals/clinicians
   ☐Patients/caregivers/people with lived or living experiences
   ☐Indigenous communities
   ☐Not-for-profit/charities Others (please specify)
   ☐Other: ______________________

Partnerships

13. What did the partnerships involve

	You providing information on intervention	Consultation to obtain feedback	Collaborator providing intervention	Co-designing intervention	Supporting implementation	Supporting policy change	Supporting scale and spread
Industry	☐	☐	☐	☐	☐	☐	☐
Healthcare professionals/clinicians	☐	☐	☐	☐	☐	☐	☐
Patients/caregivers/people with lived or living experiences	☐	☐	☐	☐	☐	☐	☐
Indigenous communities	☐	☐	☐	☐	☐	☐	☐
Not-for-profit/charities	☐	☐	☐	☐	☐	☐	☐
Others (please specify)	☐	☐	☐	☐	☐	☐	☐

Other activities (please specify)

____

14. When did you get to know your partners?

	Pre-existing partnership (prior to the grant)	New partnership (sparked by the grant)
Industry	○	○
Healthcare professionals/clinicians	○	○
Patients/caregivers/people with lived or living experiences	○	○
Indigenous communities	○	○
Not-for-profit/charities	○	○
Others (please specify)	○	○

15. Has eHIPP encouraged the creation of partnerships? If so, how?

16. Have your partnerships encouraged impacts? If so, how?

17. How likely do you think that the partnerships created as a part of the eHIPP will be sustainable long-term (i.e. last over at least five years)?

	Very likely	Somewhat likely	Neither likely nor unlikely	Somewhat unlikely	Very unlikely	Not applicable
With industry	○	○	○	○	○	○
With healthcare professionals/clinicians	○	○	○	○	○	○
With patients/caregivers/people with lived/living experiences	○	○	○	○	○	○
With Indigenous communities	○	○	○	○	○	○
With not-for-profit/charities	○	○	○	○	○	○

International Partnerships

18. Have international partnerships been a part of your eHII supported work?
☐No
☐Yes

19. Please tell us if the international partnerships were a part of the AAL program or arose through other channels?

20. Please share how you established your international partnerships?

21. What have been the impacts of your international partnerships? If any, how did they occur?

eHIPP Design

22. How effective has the design of the program been in terms of:

	Very effective	Somewhat effective	Neither effective nor ineffective	Somewhat ineffective	Very ineffective	Don't know
Catalyzing new e-Health innovations for improved patient and population-centered care	○	○	○	○	○	○
Fostering partnerships between health care innovation communities and industry	○	○	○	○	○	○
Increasing Canada's position in the health-related ICT industry	○	○	○	○	○	○

23. Are there any design elements of the program that have facilitated achieving impacts?

24. Are there any design elements that have hindered impacts?

25. Are there some other design elements that can potentially catalyze impacts?

Future investment in eHealth

26. Do you think there is a need for CIHR to continue to invest in digital health?

27. In your opinion, what are the current research priorities in digital health?

Evaluation

28. Please indicate your level of agreement or disagreement with each of the following statements:

	Strongly agree	Agree	Neither agree nor disagree	Disagree	Strongly disagree	Not applicable
The eHII funding initiative has been essential for my research work and my advancements in digital health over the last few years	◯	◯	◯	◯	◯	◯
I would not be able to be successful in my digital health work without my collaboration with industry	◯	◯	◯	◯	◯	◯
My collaboration with patients/caregivers/people with lived or living experiences was key to the success of my eHII work	◯	◯	◯	◯	◯	◯
My collaboration with healthcare providers/clinicians was key to the success of my eHII work	◯	◯	◯	◯	◯	◯
My collaboration with policy makers/decision makers was key to the success of my eHII work	◯	◯	◯	◯	◯	◯
The impacts of my eHII project have contributed towards health equity in Canada	◯	◯	◯	◯	◯	◯
The eHII program that supported me was effectively designed to obtain the objectives of my research	◯	◯	◯	◯	◯	◯
My international collaboration within the eHII program has had considerable impact on my success	◯	◯	◯	◯	◯	◯

29. Do you consent to CIHR featuring your Impact Narrative (below) as a part of communiques, newsletters, press releases, website entries etc.?
    ☐Yes
    ☐No

30. Optional Impact Narrative - In four short paragraphs and in the order specified (350 words or less), please provide a lay summary of the problem that needed to be addressed through your eHII innovation (first paragraph), the most important impact(s) of your work and for whom (e.g., the impacts of the eHealth innovation on cost- effectiveness, patient-centred care, and/or population care - second paragraph), how your partnerships created/fostered/enhanced/enabled your research and its impacts (your partnership process/approach[i] - third paragraph), the implications of your key findings for next steps (e.g., in policy or practice – fourth paragraph).

31. Do you have any questions to comments about this survey?

---

[i] How you worked with partners to identify the problem, address the issue, and the approach(es) to undertake the research, who else did you engage with to develop /implement /scale the intervention.
