# Supplementary material for: Research impact assessment of a Canadian digital health funding program: a case study
Source: Health Res Policy Syst. 2025 Jun 23;23:81. doi: 10.1186/s12961-025-01356-2 (PMC12183889; doi:10.1186/s12961-025-01356-2)
Supplement: Supplementary file 3 — Additional file 3. Information about the eHIPP research teams and projects. A list of the funded eHIPP projects. The table includes the key eHIPP priority area addressed, project title, and the province of the eHIPP lead investigator. [file 12961_2025_1356_MOESM3_ESM.pdf]

Table S1: Information about the eHIPP research teams and projects

Priority Population	Project Title	Province of Lead Investigator
**Older adults with complex care needs at home**	Implementation and Evaluation of an Enhanced PharmaNet-Based Adverse Drug Event Reporting Platform to Improve Patient Safety and Meet Adverse Drug Reaction Reporting Requirements	British Columbia, Canada
	Caring near and far: a multi-province investigation of remote monitoring technologies connecting community-based older adults and their care team	Ontario, Canada
	Reorganizing the Approach to Diabetes through the Application of Registries (RADAR)	Alberta, Canada
	Evaluating the Effectiveness of Integrating Patient Reported Outcomes and Assessments in the Care for Seniors with Complex Needs (e-PRO)	British Columbia, Canada
	TEC4Home: Telehealth for Emergency-Community Continuity of Care Connectivity via Home-Telemonitoring	British Columbia, Canada
	A novel approach to connecting peritoneal dialysis patients to healthcare staff-A multi-centre randomized controlled trial	Ontario, Canada
	Optimizing the health of seniors: The development, implementation, and evaluation of an electronic multi-chronic disease tool (e-MCD)	Ontario, Canada
	Delivery of self-management through a peer-support telehealth intervention in patients with cardiovascular disease: The Healing Circles Project	British Columbia, Canada
	Projet FreeDom (FreeO2 Ã domicile). Mise au point et Évaluation d'une stratégie innovante de prise en charge pour réduire la durée d'hospitalisation au cours des exacerbations de MPOC: retour précoce à domicile en combinant le sevrage et l'ajustement automatisée de l'oxygène (FreeO2) et la Télémédecine. Bénéfices pour les patients et pour le système de santé.	Quebec, Canada
	THE SMArT VIEW, CoVeRed: TecHnology Enabled monitoring and Self-Management-Vision for patient Empowerment following Cardiac and Vascular surgery	Ontario, Canada
	HIP@Home: A Community-based Monitoring, Rehabilitation and Learning e-System for patients following a Hip Fracture	Quebec, Canada
	Telemonitoring and Protocolized Case Management for Hypertension In Seniors	Alberta, Canada
	Integrating a quality-of-life assessment and practice support system in homecare services for older adults with life-limiting illness and their families	British Columbia, Canada
	Supporting Goal-Oriented Primary Health Care for Seniors with Complex Care Needs using Mobile Technology: Evaluation and implementation of the HSPRN-Bridgepoint ePRO Tool.	Ontario, Canada
**Youth with mental health conditions**	Evidence-Based e-Health Solutions for Youth with Mental Illness	Ontario, Canada
	Teacher Help: Novel technologies for meeting the immediate needs of youth with mental health disorders in Canada	Nova Scotia, Canada
	Students Mental Health: Virtual Support on Campus	Ontario, Canada
	Computer Based Training for Cognitive Behavioral Therapy (CBT4CBT) for Improved Addiction Treatment in Canadian Youth	Prince Edward Island, Canada
	An Integrated Self-Referral eHealth Strategy for Improving Rapid and Direct Access to Youth Mental Health Services: A Stepped-Wedge, Cluster Randomized Controlled Trial in Six Canadian Healthcare Settings	Quebec, Canada
	WeTakeCare: An internet patient-centered mobile-based interactive communication system to support collaborative mental health care for youth and adolescents with ADHD	Ontario, Canada
	Cognitive Behavior Therapy for Anxious and Depressed Youth: Improving Outcomes through Mobile Technology	Ontario, Canada
	ThoughtSpot: Enhancing self-efficacy for help-seeking among transition-aged youth in postsecondary settings with mental health and/or substance use concerns using crowd-sourced online and mobile technologies.	Ontario, Canada
