# Supplementary material for: Research impact assessment of a Canadian digital health funding program: a case study
Source: Health Res Policy Syst. 2025 Jun 23;23:81. doi: 10.1186/s12961-025-01356-2 (PMC12183889; doi:10.1186/s12961-025-01356-2)
Supplement: Supplementary file 4 — Additional file 4. eHealth Innovations Partnership Program – Impact Analysis Interview Guide. The eHIPP impact analysis interview guide that guided the discussion with the 13 interview participants. [file 12961_2025_1356_MOESM4_ESM.pdf]

eHealth Innovations Partnership Program – Impact Analysis

Interview Guide

Background

1. Why did you decide to apply for the eHIPP (eHealth Innovations Partnership Program) grant?

2. Who was part of your core team?

Impacts on health/health systems

3. Can you please describe your intervention briefly and tell me where it has been implemented within the health system? (For example, within a hospital, community, region, etc.).

4. Would you say your project contributed to a change in practice at your implementation site(s)?

5. Has your intervention been scaled or spread beyond the initial implementation site(s)? Please elaborate.

eHIPP's first objective was to:
Identify patient-oriented eHealth solutions that will improve health outcomes, patient experience, and lower the cost of care along the continuum for the eHIPP priority areas.

6. Do you think your project achieved the first objective of improving health outcomes, patient experience, and/or lowering the cost of care? Please elaborate on each component.

   If achieved:
   - How did you achieve these impacts? (What did you do, who did you talk to, which activities did your team pursue etc.?)

   - Were there any enabling factors and conditions that encouraged these impacts? Please elaborate.

   If not achieved:
   - What did you do to try to achieve these impacts?

   - What factors and conditions hindered impacts?

- Knowing what you know now, would you do something differently to achieve impacts?

7. Did your project contribute to changing policy in Canada?
   If so, how?

8. In your opinion have there been any broader economic and societal impacts of your eHIPP project?

Impacts on partnerships

eHIPP's second objective was to:
Foster partnerships between health care innovation communities and industry to co-develop and evaluate the clinical benefit and cost-effectiveness of eHealth solutions in clinical- and population health-environments.

9. Do you think your project fostered successful and sustainable partnerships that continued beyond the duration of the grant?

   If achieved:
   - How did these partnerships occur?

   - How would you describe your experience in working with individuals from the different sectors (i.e. with industry, patients, clinicians, policy makers)?

   - Did the partnerships contribute to enhancing the impacts of the project? Please elaborate on each of the different types of partnerships and how they contributed to the project's impacts.

   - Do you think CIHR contributed to fostering partnerships? If so, how?

   - Have you continued to work with your partners on new projects?
     If so, with which partners?

   If not achieved:

   What factors and conditions hindered the sustainability of your partnerships?

Design

Thinking more about partnerships, but now from the perspective of designing a funding opportunity.

10. What are your views about the design of the eHIPP funding opportunity in terms of requiring different partnerships (with industry, patients, clinicians, policy makers).

11. What are your views on how the eHIPP funding opportunity required matched co-funding?
    - Do you think that requiring co-funding was beneficial for your collaboration? (i.e. did it encourage your partners to be more invested in the project)

    - How challenging was it to raise the matched co-funding?

12. Did you participate in the optional partnership workshops or webinars at the beginning of the application phase? If so,
    - Although it is quite a long time ago, do you remember how effective they were?
    - Do you have any recommendations on improving these?

Evaluation

13. Do you think your project contributed in any way to make eHealth innovation more visible? If so, where, and how?

14. Do you think there has been a change in culture in the eHealth field over the last decade? For example, has it become more collaborative, better integrated. If yes, what factors contributed to the change in culture? Did the eHealth Innovations Initiative contribute to the change in culture? Please explain.

15. Where are the greatest needs for research investment in eHealth innovation or digital health innovation more broadly?

16. At this time, given the same opportunity, but with your current learnings and in the new Canadian context, how would you re-design a new project that promotes Canadian e-health innovations?
